# “Let Me Hear Your Handwriting!” Evaluating the Movement Fluency from Its Sonification

**DOI:** 10.1371/journal.pone.0128388

**Published:** 2015-06-17

**Authors:** Jérémy Danna, Vietminh Paz-Villagrán, Charles Gondre, Mitsuko Aramaki, Richard Kronland-Martinet, Sølvi Ystad, Jean-Luc Velay

**Affiliations:** 1 Laboratoire de Neurosciences Cognitives, LNC, UMR 7291, FR 3C FR 3512, CNRS—Aix-Marseille Université, 13331 Marseille Cedex 3, France; 2 Laboratoire de Mécanique et d’Acoustique, LMA, CNRS, UPR 7051, Aix-Marseille Univ, Centrale Marseille, F-13402 Marseille Cedex 20, France; VU University Amsterdam, NETHERLANDS

## Abstract

The quality of handwriting is evaluated from the visual inspection of its legibility and not from the movement that generates the trace. Although handwriting is achieved in silence, adding sounds to handwriting movement might help towards its perception, provided that these sounds are meaningful. This study evaluated the ability to judge handwriting quality from the auditory perception of the underlying sonified movement, without seeing the written trace. In a first experiment, samples of a word written by children with dysgraphia, proficient children writers, and proficient adult writers were collected with a graphic tablet. Then, the pen velocity, the fluency, and the axial pen pressure were sonified in order to create forty-five audio files. In a second experiment, these files were presented to 48 adult listeners who had to mark the underlying unseen handwriting. In order to evaluate the relevance of the sonification strategy, two experimental conditions were compared. In a first ‘implicit’ condition, the listeners made their judgment without any knowledge of the mapping between the sounds and the handwriting variables. In a second ‘explicit’ condition, they knew what the sonified variables corresponded to and the evaluation criteria. Results showed that, under the implicit condition, two thirds of the listeners marked the three groups of writers differently. In the explicit condition, all listeners marked the dysgraphic handwriting lower than that of the two other groups. In a third experiment, the scores given from the auditory evaluation were compared to the scores given by 16 other adults from the visual evaluation of the trace. Results revealed that auditory evaluation was more relevant than the visual evaluation for evaluating a dysgraphic handwriting. Handwriting sonification might therefore be a relevant tool allowing a therapist to complete the visual assessment of the written trace by an auditory control of the handwriting movement quality.

## Introduction

Many human actions generate sounds, whose time variations directly inform about the dynamic characteristics of the movements made. When these actions are frequently produced, the action and the associated auditory perception are bound to provide a unified percept [1–3]. Once such multimodal representation is created, it is possible to evoke the movement simply by its associated sound. Even if all the underlying brain mechanisms are not known, close connections between auditory and motor areas may explain these strong audio-motor associations [4–8]. In addition, multimodal neurons coding for both movements and their auditory consequences are known to exist within premotor brain areas [9]. In particular, it has been shown that mirror neurons in the monkey ventral premotor cortex discharge when the animal performs a specific action, but also when it hears the corresponding action-related sound without seeing the action in question [10]. The same observation has been made in Human beings [11].

However, some movements are also achieved in silence: they don’t provoke any sound or only a slight sound. When an action is naturally silent, adding sounds allows alternative perceptions and new insights about this action. In particular, associating sounds with “hidden” variables of the movement can make them perceptible for an external examiner or for the person producing the movement. This so-called movement sonification represents an approach to enrich movement perception by adding an auditory component to the kinematic or dynamic movement parameters [12]. Auditory perception of sonified movements has been shown to be effective in motor control and learning [8, 13–19]. The multisensory integration of sonified movement seems to improve both their perception and reenactment [12, 19, 20]. In consequence, the sonification of silent movements can be exploited to create a more stable and accurate cognitive representation of the movement and then be of great interest for facilitating and improving motor learning [8, 12].

Handwriting learning is one of the most difficult and long-lasting motor learning processes, which often presents significant challenges for many children. It is worth noting that the graphic movement is not the central target of handwriting teaching at school, the ultimate goal of handwriting being to produce a legible trace on a sheet of paper. As a matter of fact, the quality of handwriting is judged by its visual trace and teachers inform the children about the correctness of their handwriting by giving them posteriori feedback about the final product, the visual trace, and generally not about the ongoing process, i.e. the handwriting movement. Consequently, the diagnosis of poor handwriting, or dysgraphia, is based on the evaluation of the legibility of the written trace rather than the movement that generated it. Yet, while some children produce illegible handwriting and are easily identifiable as dysgraphic, others produce a relatively readable trace that fails to convey any problem other than a certain slowness and a difficulty in writing [21]. Thus, if kinematics could become easily perceivable through an exteroceptive sensory modality, it would be very useful for the diagnosis and the rehabilitation of dysgraphia [22]. Therefore, the main goal of this study is to translate handwriting movements into sounds to make them perceptible.

Until today, very few experiments have been done to test the effects of handwriting movement sonification. The first attempts were carried out a few decades ago in handwriting rehabilitation, mainly as a means of compensating disabilities in other sensorimotor deficits. For example, auditory feedback was applied for treatment of writer’s cramp [23, 24]. The method consisted of transforming Electromyography (EMG) recording during handwriting into auditory biofeedback in order to help the writers improve their control of excessive muscle contractions. Although the method seemed attractive, criticisms and methodological issues about these studies were reported [25]. In particular, handwriting involves contracting numerous small muscles that are not easily reachable with surface EMG. Rather than applying auditory feedback linked to the muscle activity, Baur et al. [26] proposed to sonify the grip force of the fingers onto the pen. The auditory feedback consisted of a continuous low-frequency tone when the average grip force exceeded 5 N. The tone frequency increased in four steps depending on the grip force level and patients with writer’s cramp were instructed to perform the writing exercises in such a way that they heard a pleasant, low-frequency, tone. After seven hours of training, the grip force and the pressure applied by the pen on the paper decreased but the fluency of their handwriting did not change significantly. These results were therefore encouraging for the rehabilitation of writer’s cramp but not for handwriting learning. More recently, Plimmer et al. [27] used auditory feedback together with a haptic feedback to help blind children in learning how to sign. This multimodal system translated digital ink from the teacher’s stylus gestures into two non-visual feedbacks: audio pan and pitch represented the x and y movement of the stylus; kinesthetic information was provided to the child through a force-feedback haptic pen that mimicked the teacher’s stylus movement. The authors concluded that this multisensory feedback was efficient, but they did not supply a precise analysis of the kinematic variables and the specific effect of the auditory feedback itself was not tested.

A simple way of sonifying handwriting movement is to record the displacement of the pen on the surface of a graphic tablet and to transform the (x,y) coordinates of the pen position into relevant kinematical variables which can in turn be sonified. The main question is: what are the relevant variables to sonify and which sounds should be associated to them? Danna, Paz-Villagrán and Velay [28] reported the most frequently studied variables in 42 studies aiming to characterize graphic movement disorders. They noticed that poor handwriting is characterized by long-lasting pen-lifts [29], many stops [30], a dysfluency (a lack of smoothness) [28, 31] and, sometimes, an inappropriate vertical pressure of the pen on the paper [32]. Finally, as regards the incorrectness in handwriting kinematics, they concluded that two types of variables seem particularly informative: speed and dysfluency.

Once the relevant variables have been selected, the final step is to select the sonification strategy that consists of defining the sets of mapping between data and auditory dimensions. Several strategies are possible for movement sonification [17, 20, 33]. The first strategy here consisted of associating an ‘auditory alarm’ with given ‘hidden’ incorrect variables of the movement to reveal them. The second strategy consisted of using an intuitive mapping between sound and movement. It is clear that the effectiveness of auditory feedback depends heavily on the intuitiveness and correctness of the interpretation of the applied mapping functions and metaphors. Consequently, the sonification has to respect the relevant production rules; otherwise the generated sound can lead to misinterpretations of the action [34].

How can an intuitive mapping between sound and handwriting movement be created? If we listen carefully to the sound produced during handwriting, we can hear a friction sound generated by the pen-paper interaction, especially when the surface is rough. The friction between the pen tip and the paper asperities produce timbre variations in the sound related to the handwriting kinematics that may, to a certain extent, enable the recognition of some movement characteristics produced by the writer.

In line with such assumptions, a recent and important contribution has been supplied by Thoret et al. [35]. The authors used a synthetic friction sound whose timbre variation was related to the pen velocity in two experiments. In a first experiment, subjects listened to the pen friction sounds without seeing the corresponding trace and they were asked to adjust the sounds so that they evoked a “natural” and “fluid” graphical movement. Results showed that they adjusted the timbre variations that were directly linked to the velocity profile according to the 1/3 power law [36, 37]. In other words, the timbre variations of the sound of a moving pen appear to vary in accordance with the kinematic rule governing real graphical movements. They concluded that timbre variations generated by a velocity profile that obeys the 1/3 power-law evoke a natural and fluid biological movement. This finding demonstrates that sounds can evoke a gesture and adequately inform about drawing movements if their acoustic characteristics are in accordance with the kinematic rule governing actual movements. In a second experiment, the authors investigated our ability to identify drawn shapes. Subjects were asked to associate friction sounds with simple graphic shapes (circle, ellipse, arches, line, lemniscate, loops). They concluded that discriminating between visual shapes on the basis of their produced sounds is, therefore, possible if the acoustic characteristics of the sounds differ sufficiently.

However, these results were acquired with simple graphic shapes (circle, ellipse…) which were drawn by the very fluid movements of an adult writer. Contrary to drawing simple shapes, handwriting imposes complex movements which are not always fluid, in particular for children who are learning to write. In handwriting, the pen slows down and accelerates sometimes independently from the curvature of the drawn shape. Since timbre variations afford the action of drawing fluidly and naturally, would it be possible to deduce the quality of the underlying handwriting movements only by hearing the friction sounds they generate?

Our aim was to identify acoustic cues that reflect the movements underlying handwriting and to ascertain the extent to which this information allows the listener to deduce the fluency of handwriting. If the mapping of the kinematical variables to the sounds is relevant, it should be possible to evaluate the handwriting quality only by hearing it (without seeing it), and without any information about what is heard (i.e. implicitly).

Three experiments were performed. In a first experiment, samples of handwriting from members of the three categories of writers (children with dysgraphia, children with proficient handwriting, and adults with proficient handwriting) were collected and analyzed. Then, these samples were sonified to create audio files providing the material for the auditory experiment (Experiment 2), and printed to create visual stimuli for the visual experiment (Experiment 3). In a second experiment, the audio files were presented to 48 listeners who had to mark the quality of the underlying unseen handwriting. In a first condition called ‘implicit’, they first made their judgment without any knowledge of the exact mapping between the sounds and the handwriting variables. They just knew that they were hearing handwriting. From a purely practical point of view, the implicit situation was not justified since it will never occur in real life: Anyone judging handwriting quality from the associated sounds would know the sound/variable mapping. Nevertheless, even if it is somewhat artificial, this situation is interesting and important because it allowed us to assess whether the mapping we used was intuitive for the listeners. For the sake of efficiency, it was important not to use a counterintuitive mapping to avoid undesirable percepts and unexpected results [34]. In a second condition called ‘explicit’, listeners made their judgments with some information about the meaning of the sounds and the evaluation criteria.

Finally, in a third control experiment, the written words were visually presented to 16 new participants who had to mark the quality of the handwriting. Importantly, during the whole experiments 2 and 3, the participants were unaware that the handwriting they heard or saw belonged to three categories of writers.

## Experiment 1: Handwriting Collection

### Materials and Methods

#### Participants

Fifteen writers, divided in three groups, volunteered for the experiment. The first group was composed of five children with dysgraphia (DC) in grade 3 (10.8 years ± 0.6 years, 1 girl), the second group of five proficient children (PC) in grade 3 (8.2 years ± 0.3 years, 2 girls), and the last group of five proficient adults (PA) without known language or motor impairments (32 years ± 2.5 years, 3 women). The children with dysgraphia had been diagnosed by means of the Concise Assessment Scale for Children’s Handwriting [38, 39]. The study had a prior approval by the Ethics Committee of the Aix-Marseille University and CNRS (N° RCB 2010-A00155-34). The adult participants signed a written informed consent after the procedure was fully explained. For all children, parents signed an informed consent prior to the experiment.

#### Task

The task consisted of writing the French word ‘*lapin*’ (rabbit) eight times, at a spontaneous velocity, and in cursive letters, on a paper fastened on a graphic tablet (Wacom, Intuos3 A4, sampling frequency 200Hz) using an ink pen. This word was used because it is a familiar word without any spelling difficulty. Participants had to write the word inside eight rectangles (5.0 x 1.5 cm) that were drawn on the paper sheet. The model was first shown to the writers to ensure that they knew the spelling of the word. For both data acquisition and processing, we used a program developed in the laboratory which discriminates and visualizes both drawn and ‘in air’ movements (between letters) during handwriting.

#### Data analysis

The eight repetitions of the written word were segmented into different parts based on the pen contact on the tablet (null pressure). Because the moment at which subjects put the dot on the letter ‘*i*’ varies greatly both within and between writers, we decided to remove this point and the two ‘in air’ movements preceding and following it.

Three kinematic variables were analyzed: the instantaneous translational velocity, the dysfluency and the rate (for the sake of clarity, we adopted here the term of rate rather than frequency which can be mistaken with sound frequency). The instantaneous translational velocity was determined by computing the difference between the pen’s coordinates divided by the time difference between two successive positions. The dysfluency movement was characterized by the variable *Signal-to-Noise velocity peak difference* (SNvpd) [28]. This variable was calculated as follows: the instantaneous translational velocity was filtered using two filters at different cutoff frequencies: the first one at 5 Hz to determine the normal fluctuations, i.e. those induced by changes in the curve of the required trajectory [37], and the second one at 10 Hz in order to determine the real movement fluctuations. By subtracting the velocity peaks obtained when filtering with a cutoff frequency of 5 Hz (due to normal fluctuation) from those obtained when filtering with a cutoff frequency of 10 Hz, the ‘abnormal’ velocity peaks, corresponding to neuromotor noise [31], were localized and counted (for an illustrated example, see [28]). The peak difference was small in fluent handwriting movements and large in jerky, non-fluent movements. Lastly, the rate of a handwriting movement corresponded to the mean number of cycles written per second. For example, if the letter ‘*e*’, requiring two half-cycles (two velocity peaks), is written in one second, the corresponding rate of handwriting is equal to 1 Hz.

Two temporal variables were also analyzed: the Movement Time (MT) corresponding to the time required to write the entire word, and the percentage of pen lift duration, corresponding to the time during which the pen was not in contact with the paper. Finally, one spatial variable was analyzed: the trace length, corresponding to the total distance traveled by the pen while in contact with the sheet.

For each writer, the six variables were averaged across the eight repetitions and submitted to a non-parametric repeated measures ANOVA (Kruskal-Wallis) with AD, PC, and DC as group factors and completed with non-parametric multiple comparisons. These comparisons among pairs of groups were done by means of the Mann-Whitney U test for two independent samples. The p threshold for multiple comparisons was corrected using the Bonferroni method (differences of p < 0.016 were considered to be statistically significant for the three pairwise analyses).

### Results

The raw data are within the Supporting Information file S1 Dataset. Individual performances are presented in Table 1. The Kruskal-Wallis tests revealed a main Group Effect on velocity (H(2, 15) = 7.62, p < 0.05, *η*² = 0.49), on rate (H(2, 15) = 12.52, p < 0.001, *η*² = 0.83), on dysfluency (H(2, 15) = 10.39, p < 0.01, *η*² = 0.83), on percentage of pen lift duration (H(2, 15) = 6.03, p < 0.05, *η*² = 0.49), on MT (H(2, 15) = 12.5, p < 0.001, *η*² = 0.73), and on trace length (H(2, 15) = 9.42, p < 0.01, *η*² = 0.49). P-values for the multiple comparisons tests are presented in Table 2.

**Table 1:** Mean kinematic characteristics for the written word ‘*lapin*’ for each writer and for each group, i.e. Children with Dysgraphia (DC), Proficient Children (PC) and Proficient Adults (PA) group.

Group	Writer	Velocity (mm/s)	Rate (Hz)	Dysfluency (SNvpd)	Pen lift duration (%)	MT (s)	Trace Length (mm)
**DC**	AN	28	2.54	5	42.3	6.32	109
	FL	15	2.30	23	2.2	6.21	87
	JU	29	2.24	13	34.7	6.87	123
	ME	25	2.64	4	27.0	4.60	85
	NA	22	2.32	18	8.4	7.08	137
	Mean	23.7	2.41	12.6	22.9	6.21	108.5
**PC**	CO	41	2.71	4	9.4	2.97	108
	ET	36	3.05	3	12.7	2.87	89
	JE	48	2.89	0	17.9	3.62	139
	MO	28	2.72	3	22.3	4.35	93
	SA	32	2.89	3	15.4	3.22	81
	Mean	36.9	2.85	2.6	15.6	3.41	102.5
**PA**	AU	33	4.35	1	4.7	1.83	56.5
	DJ	24	3.27	1	0	2.40	58.3
	JC	27	4.00	2	0	1.87	51.0
	LU	30	4.34	2	9.8	2.04	55.6
	JO	30	3.53	1	7.0	2.14	59.4
	Mean	28.9	3.90	1.4	4.3	2.06	56.2

**Table 2:** *P*-values for each of the Mann-Whitney U tests for multiple comparisons between groups, i.e. Children with Dysgraphia (DC), Proficient Children (PC) and Proficient Adults (PA) group.

Group comparison	Velocity	Rate	Dysfluency	Pen lifts duration	MT	Trace Length
**DC vs PC**	0.015	0.008	0.008	NS	0.008	NS
**DC vs PA**	NS	0.008	0.008	NS	0.008	0.008
**PC vs PA**	NS	0.008	NS	0.015	0.008	0.008

NS: not significant.

### Discussion

The results of this pre-experiment confirmed that writing a simple isolated word is sufficient to reveal significant differences in performance between the three groups of writers. The time required to write the word differs between the three groups. These differences are explained by a lower velocity in DC compared to PC, a higher dysfluency in DC compared to the other two groups, a different rate between the three groups, a lower percentage of pen lift duration in PA compared to PC, and a lower trace length in the adult group compared to the two other groups. Note that the results did not reveal significant differences between the DC group and the two other groups in the pen lifts duration due to a very variable performance within the DC group.

An interesting observation is that PC were not systematically slower than adults. Some PC were even faster than certain adults. However, handwriting rate was higher in the adults. Consequently, the lower total MT in adults resulted from a faster rate and not from the velocity of their writing. This increase in rate was reached by reducing the letter size and thus by writing letters in less time without going more quickly, strictly speaking. Note also that individual performances within the DC group were heterogeneous: Some children wrote slowly, others wrote with a dysfluency, or they lost time during pen lifts. In conclusion, all the variables analyzed here revealed at least one significant difference in multiple comparisons. At this point, the question was how to choose the variables to sonify and how to map them onto sounds that would offer the most appropriate information about the handwriting quality.

## Handwriting Sonification

Two different strategies of sonification were applied. The first one consisted of using a natural mapping between the sound and the pen velocity in order to contribute to the building of a multimodal sensorimotor representation of handwriting. The sonification strategy was defined so that the sounds might be intuitive for the listeners: we chose the evocation of friction sounds that were naturally produced by a writer with the pen on a sheet of paper. We used the friction sound synthesis model previously used by Thoret et al. [35]. This model simulates the physical sound source as the result of successive impacts of a pencil on the asperities of a given surface. The surface roughness is modeled by a noise reflecting the height of the surface asperities while the velocity profile of the pencil is modeled by low-pass filtering the noise with a time varying cutoff frequency that creates timbre variations according to the velocity profile. The sounds generated with this model have been compared to real recorded pen friction sounds and they evoked the same perception [35]. Furthermore, the pen pressure on the paper sheet, a measure directly provided by the tablet in non-scaled units from 0–1023, was linearly associated to the sound volume, so that the higher the pen pressure the higher the volume. To avoid too great differences in volume between all trials, the pressure was first normalized by dividing the pressure values by the maximal value for each trial. During pen lifts, no sound was emitted and hence the duration of silences directly informed about pen lifts duration.

The second sonification strategy was different and used for movement dysfluency which is an artificial index of movement quality (abnormal velocity fluctuations). The aim was to inform about a break in movement fluency by means of a discrete sound playing the role of an ‘auditory alarm’. In practice, when the handwriting was jerky, i.e. for *each supernumerary velocity peak* determined by the SNvpd, a sound was emitted. The velocity peaks are obviously inaudible in a natural situation and have therefore never been heard before. They were sonified by very short crackling sounds, similar to the crackling of vinyl records which were used as a metaphor: the better the handwriting movement, the fewer the crackling sounds. These impact sounds were created using the synthesizer developed by Aramaki and colleagues (for more details, see [40]). Their duration was about 15ms.

## Experiment 2: Handwriting Listening

### Materials and Methods

#### Participants

Forty-eight adults (28.8 years ± 6.7 years, 24 women), recruited among the staff and the students of the laboratory volunteered for the experiment. They were not experts in handwriting and they were fully naïve as regard the aim of the experiment. They had not been involved in the previous handwriting collection experiment. None of them reported known language or auditory impairment.

#### Stimuli

From the eight writing repetitions of experiment 1, we built eight audio files. However, as can be seen in Table 1 (MT values), writing the same word took almost twice as long in PC and three times longer in DC than in PA. Therefore, not all the audio files corresponding to a given instance of the word were of the same duration. This duration difference would have made the writings of children and adults too easy to differentiate between and would have biased the perceptual judgment. To make all the audio files of the same duration (corresponding to the DC handwriting, 6.2s), we concatenated 2 different files from the same writer of the PC group, and 3 different files from the same writer of the PA group. We did not concatenate the same file two or three times to avoid a possible repetition effect with the same pattern. The concatenated files were randomly chosen among the 8 repetitions with the sole constraint that the final duration of the audio file was as close as possible to 6.2s. The mean duration of the final audio files was 6.2s (± 1.2s), 6.7s (± 1.2s) and 6.2s (± 0.6s) for DC, PC and PA groups respectively.

Three of the audio files are available with the electronic version of the paper. These files correspond to prototypal sonified handwriting of one writer of each group (a child with dysgraphia—S1 Audio, a child with a proficient handwriting—S2 Audio, and an adult with a proficient handwriting—S3 Audio).

#### Task

Participants were informed that they would have to mark the quality of an example of cursive handwriting on a scale from 0 to 20 without seeing it, thus basing their marks solely on the sounds they heard. One third of the sounds (15) corresponded to the sonified handwriting of DC, one third to that of PC and the last third to that of PA. They were not told during the whole experiment that they would hear the handwriting of three different categories of writers. They were equipped with headphones in a quiet room. They listened to the audio files and had to mark them one by one using a paper sheet. The experiment consisted of three conditions, always presented in the same following order: a “training” condition, an “implicit” condition and an “explicit” condition. The training condition was conceived to familiarize the listeners with the entire experimental procedure, the sounds associated to the handwriting and the grading procedure. During the training, participants were asked to mark 18 sounds (6 for each category of writers) chosen among the 45 files. At no time, was the meaning of the sounds, that is the association principles between the kinematical characteristics of handwriting movements and sounds, explained to the participants. During training, all listeners had exactly the same 18 files in the same order and they were given no feedback about the validity or relevance of their marking. The “implicit” condition was identical to the training condition, but all the 45 experimental sounds were presented. At this step, no additional information was given to listeners about the meaning of the sounds. Finally, after the ‘implicit’ and before the ‘explicit’ conditions, the participants were provided with the following information:

- The continuous sound corresponds to the handwriting speed. Fast speed and regular rate should be considered as good handwriting,
- Silences correspond to pen lifts. Good handwriting has few pen lifts.
- The brief crackling sounds correspond to handwriting ‘saccades’ and dysfluency. Fluent handwriting, with few cracking sounds, should be considered as good.

This information was written on the notation sheet and present throughout the explicit condition. Participants had to use it to evaluate the auditory trials.

The 45 audio files in the implicit and explicit conditions were distributed in a pseudo-random order for each listener in such a way that two audio files of a same group of writers were not placed successively for the implicit and for the explicit conditions. The experiment lasted for about one hour.

#### Data analysis

Two independent variables were controlled: “Knowledge” (implicit vs. explicit) and “Category” of writers (DC, PC and PA). In both the ‘implicit’ and ‘explicit’ conditions, and for each listener, the mean score per category of writers was computed from 15 trials corresponding to the 3 audio files of the 5 writers belonging to this category. A repeated measures ANOVA (type III) was carried out with these mean scores. Bonferroni’s post-hoc tests were used when necessary. Then, a cluster analysis was conducted on the individual scores in the implicit condition. The scores were analyzed by interactive partitioning (K-means), minimizing the within-cluster variability and maximizing the between-cluster variability. Finally, in order to determine the relative contribution of each variable, multiple linear regression between the scores and the kinematic characteristics of each trial (see Table 1) was analyzed.

### Results

The scores given by the listeners are within the Supporting Information file S2 Dataset.

#### Analysis of variance

**Fig 1:**
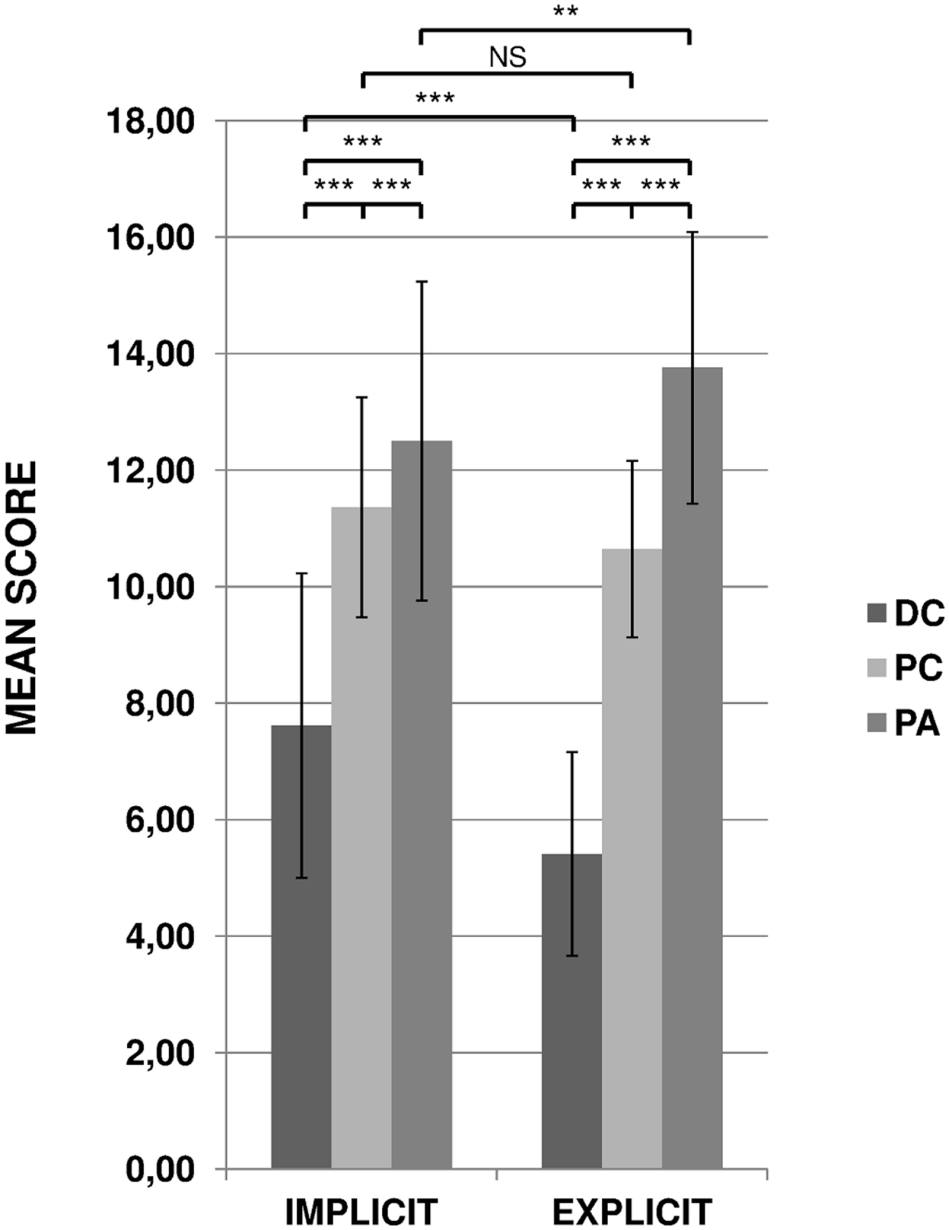
Mean scores given by the 48 listeners for Children with Dysgraphia (DC), Proficient Children (PC) and Proficient Adults (PA) under the implicit and explicit conditions. Error bars correspond to inter-individual variability. NS: not significant; **: p< 0.01; ***: p < 0.001.

The ANOVA revealed a main effect of “Knowledge” (F(1, 47) = 6.45, p < 0.05, *η*_G_² = 0.02). Results are presented in Fig 1. As a whole, the mean scores were lower in the ‘explicit’ than in the ‘implicit’ condition. The “Category” factor was also significant (F(2, 94) = 166.91, p < 0.0001, *η*_G_² = 0.62). The post-hoc tests revealed that the scores attributed to the three categories of writers were significantly different, both under ‘implicit’ and ‘explicit’ conditions. Finally, the “Knowledge” by “Category” interaction was significant (F(2, 94) = 28.79, p < 0.0001, *η*_G_² = 0.10). As can be seen in Fig 1, the between group differences were greater in the ‘explicit’ than in the ‘implicit’ condition. Even though the scores attributed to PC did not greatly change from one condition to the other, the DC scores decreased whereas the PA scores increased.

#### Cluster analysis in implicit condition

In a second analysis, we were interested to find out whether the significant effects revealed by the ANOVA corresponded to a general trend in all listeners or if it was a difference of strategy in some listeners in the implicit condition. We thus conducted a cluster analysis on the individual scores in the implicit condition. Initially, we wanted to separate the participants who succeeded in implicitly discriminating the 3 categories of writers (cluster 1) from those who did not (cluster 2). The cluster analysis of the implicit condition confirmed the presence of 32 participants in cluster 1 and 16 in cluster 2. Then, when we carried out the correlation analyses to find out what the variables used to achieve the perceptual judgment were, it so happened that none of the correlations were significant in cluster 2. We suspected that this absence of correlation was the consequence of opposite correlations among the participants belonging to cluster 2. Therefore, we decided to separate the participants into three clusters. The cluster analysis of the implicit condition confirmed the presence of three clusters with inter-cluster Euclidian distances of 3.56 between clusters 1 and 2, of 3.09 between clusters 2 and 3, and of 3.56 between clusters 1 and 3. The first cluster included 32 (66%) participants, the second included 10 (21%) participants, and the third the remaining 6 (13%). We then submitted the results of the ‘implicit’ situation to an ANOVA with ‘Listeners Cluster’ as Group factor and ‘Writers Category’ as repeated measures. This analysis revealed a main effect of ‘Listeners Cluster’ (F(2, 45) = 18.45, p < 0.0001, *η*_G_² = 0.21), a main effect of ‘Writers Category’ (F(2, 90) = 19.88, p < 0.0001, *η*_G_² = 0.23), and a ‘Listeners Cluster’ by ‘Writers Category’ interaction (F(4, 90) = 27.87, p < 0.0001, *η*_G_² = 0.45).

**Fig 2:**
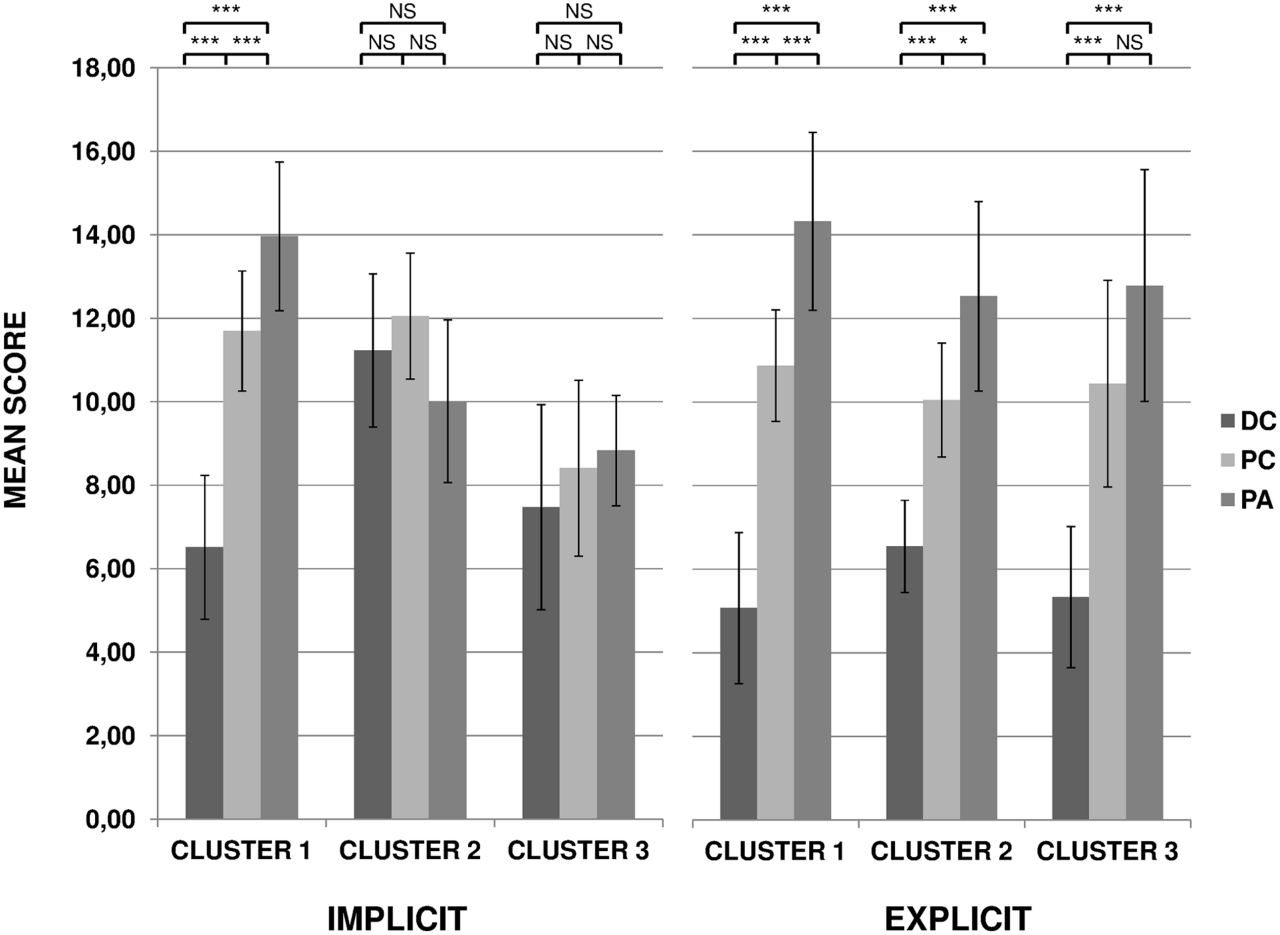
Mean scores given by the three clusters of listeners for Children with Dysgraphia (DC), Proficient Children (PC) and Proficient Adults (PA) in implicit (left) and explicit (right) conditions. Error bars correspond to inter-individual variability. NS: not significant; **: p< 0.01; ***: p < 0.001.

The post-hoc analysis of the ‘Listeners Cluster’ effect revealed that the mean score given by cluster 3 (8.24/20) was significantly lower than the mean scores of clusters 1 and 2 (10.72 and 11.09, respectively). The post-hoc analysis of the ‘Writers Category’ effect revealed that, as a whole, the 3 categories of writers were not scored identically. More interestingly, the post-hoc analysis of the interaction revealed that the listeners belonging to cluster 1 clearly discriminated between the three categories of writers (p < 0.0001) whereas those of cluster 2 and 3 did not (Fig 2, left). To sum up, two thirds of the listeners (32/48) succeeded in significantly discriminate between the three categories of writers, although they had no information about the presence of three categories of writers, nor about the meaning of the sounds they heard. Finally, clusters 1 and 2 had in common a mean score close to each other and different from cluster 3 and clusters 2 and 3 shared the same behavior: they did not mark the 3 writer categories differently.

In order to interpret each cluster and understand the evaluation criteria used to score the handwriting, the same clusters were compared in the “explicit” condition. The ANOVA with Listeners Cluster as Group factor and Writers Category as repeated measures showed a main effect of Writers Category (F(2, 90) = 195.04, p < 0.0001, *η*_G_² = 0.66). As can be seen in Fig 2 (right part), the three writers groups were clearly separated into three clusters. However, the ‘Writers Category’ by ‘Listeners Cluster’ interaction was still significant (F(4, 90) = 5.07, p < 0.001, *η*_G_² = 0.09). The post-hoc analysis of the interaction revealed that the listeners belonging to cluster 1 and 2 did discriminate between the three categories of writers (Fig 2, right), with a slightly less pronounced discrimination in cluster 2 than in cluster 1. However, listeners belonging to cluster 3 only discriminated between the dysgraphic handwriting and the two other writer categories, which did not differ significantly (p = 0.30).

#### Multiple linear regression analysis

Finally, we wanted to know which sonified variable (instantaneous speed, supernumerary velocity peaks or instantaneous pressure) contributed the most to the discrimination judgment. In order to determine the relative contribution of each variable, multiple linear regression analysis between the scores and the kinematic characteristics of each trial (see Table 1) was conducted for each cluster and each condition (implicit and explicit). In all, the multiple linear regression analysis between the given scores and the following four variables was conducted: velocity, rate, dysfluency (supernumerary velocity peaks), and percentage of pen lift durations. Overall results are summarized in Table 3.

**Table 3:** Report of the beta-weights of the significant regression between the scores given by the listeners of each cluster and the corresponding kinematic characteristics of all audio files in implicit and explicit conditions.

	Implicit condition			Explicit condition		
	Cluster 1	Cluster 2	Cluster 3	Cluster 1	Cluster 2	Cluster 3
Velocity (mm/s)	0.22	NS	NS	0.23	NS	0.15
Rate (Hz)	0.17	-0.24	NS	0.33	0.21	0.22
Dysfluency (SNvpd)	-0.29	NS	NS	-0.27	-0.41	-0.40
Pen lift duration (%)	-0.37	NS	-0.17	-0.34	-0.28	-0.19

NS: not significant.

*Implicit condition*, *Cluster 1*: The analysis revealed a significant regression (F(4, 1435) = 329.21, p < .0001, R² = 0.48). The regression tests confirmed that the four correlated variables contributed to the prediction of the 1440 scores (45 trials × 32 listeners) given by the listeners of Cluster 1 in the implicit condition (p < .0001 for the four variables, see Table 3 for beta-weight values). The scores were negatively correlated with the dysfluency (SNvpd) and with the percentage of the pen lift duration, and positively correlated with rate and velocity.

*Implicit condition*, *Cluster 2*: The analysis revealed a significant regression (F(4, 445) = 7.70, p < .0001, R² = 0.06). The regression tests revealed that only the rate contributed to the prediction of the 450 scores given by the listeners of Cluster 2 (p < .01, see Table 3). Therefore, for Cluster 2, the correlations between variables and scores were weak and the only variable (rate) that predicted the score had a reverse sign with respect to Cluster 1.

*Implicit condition*, *Cluster 3*: The analysis did not show a significant regression (F(4, 265) = 1.54, p = .19, R² = 0.02), however the regression tests showed that the percentage of pen lift duration slightly contributed to the prediction of the scores (p < .05, see Table 3). The 270 scores (45 trials x 6 listeners) given by the listeners were only negatively correlated with the percentage of pen lift duration. The six listeners of Cluster 3 only seemed to have considered the lifts (i.e. the silences) when judging the handwriting quality.

*Explicit condition*, *Cluster 1*: The analysis revealed a significant general regression (F(4, 1435) = 610.13, p < .0001, R² = 0.63). As in the implicit condition, the regression tests confirmed that the four correlated variables contributed to the prediction of the scores (p < .0001 for the four variables, see Table 3). The same correlations as found in the implicit condition were also observed in the explicit condition. Therefore, for listeners of Cluster 1, providing the meaning of the sounds did not give rise to qualitative changes in their judgment, but only to slight quantitative changes.

*Explicit condition*, *Cluster 2*: The analysis revealed a significant regression (F(4, 445) = 100.56, p < .0001, R² = 0.47), the regression tests showing that the dysfluency, the percentage of pen lift duration, and the rate contributed significantly (p < .0001 for the three variables, see Table 3), and that the velocity contributed marginally (p = .07), to the prediction of the scores. The 450 scores given by the listeners were correlated to the same variables as those given by the listeners of Cluster 1. Therefore, for Cluster 2, explaining the meaning of the sounds gave rise to qualitative changes in the way they scored.

*Explicit condition*, *Cluster 3*: The analysis revealed a significant regression (F(4, 265) = 61.23, p < .0001, R² = 0.47) and the regression tests confirmed that the four correlated variables contributed to the prediction of the scores (p < .0001 for the dysfluency and for the percentage of pen lift; p<.01 for the rate; p <.05 for the velocity, see Table 3).

To sum up, as soon as the listeners of Cluster 2 and 3 were informed about the meaning of the sounds and the evaluation criteria, they changed their evaluation to respect the instructions. Interestingly, the listeners of the three clusters gave a lower score to the performances of children with dysgraphia than to the two other writer groups.

### Discussion

#### Explicit condition

As a whole, the results showed that the participants were able to perform the evaluation task efficiently. As a matter of fact, in the explicit condition, the listeners of the three clusters were able to differentiate the poor handwriting of children with dysgraphia from the fluent handwriting produced by proficient children and adults (see Fig 2). Whereas one third of listeners (those belonging to clusters 2 and 3) did not correctly interpret the meaning of the sounds implicitly, the explanations of the meaning of sounds and of evaluation criteria improved their evaluation and led them to differentiate significantly between proficient and dysgraphic handwriting. This result suggests that the auditory evaluation of sonified handwriting is a skill both quickly and easily acquired.

Which were the most important variables for the perceptual judgment? Under the explicit condition, the correlations between handwriting variables and the scores of all the listeners suggested that they all were used for judging the quality of the heard handwriting: Velocity was positively correlated (0.41) as well as rate (0.65), while dysfluency was negatively correlated (-0.58) as well as the pen lift duration (-0.39). The regression analysis confirmed that all the variables played a significant role, but the correlations with scores of correctness of handwriting were greater for dysfluency and rate.

The dysfluency and the mean velocity were not statistically different between PC and PA, whereas rate was greater in PA and pen lift duration lower in PA than in PC (Tables 1 and 2). We think that the silences (corresponding to pen lifts) and, in particular, the continuous friction sound (corresponding to instantaneous translational velocity) were sufficient to evaluate them differently. Sonifying instantaneous translational velocity informs about both mean velocity and rate. Our results showed that the rate seemed more relevant than the mean velocity itself for judging of the quality of handwriting. This difference in rate, might explain the higher scores of PA.

Overall, the differences between PC and DC were greater than those between PC and PA. In other words, it was easier for listeners to evaluate negatively poor handwriting (DC) than to positively evaluate a good one (PA). We think that this might be the consequence of sonifying the dysfluency, which was much more pronounced in DC than in the two other groups that did not significantly differ. As a matter of fact, translating supernumerary velocity peaks into discrete sounds made them easily identifiable and quantifiable. On the other hand, differences of rate between PC and PA were revealed by differences in the continuous friction sound that were probably more difficult to perceive.

#### Implicit Condition

Although the sonification model and mapping we used was quite natural and had already been validated in a previous experiment [35], except for the brief crackling sounds associated to the supernumerary velocity peaks, we assumed that it was important to check this point. The results showed that in the implicit condition, two thirds of the listeners (the 32 belonging to cluster 1) marked the three categories of writers differently. Although they did not know the meaning of the sounds, they interpreted them correctly. The correlations between variables and scores were less but the same as in the explicit condition. Therefore, the sonification mapping seemed to be quite intuitive for the majority of the subjects.

Although handwriting is not really a noisy activity, the weak sounds generated by the pen friction on the paper can be heard in a silent environment, especially when the surface is rough (e.g. during writing with the chalk on the blackboard). Consequently, these sounds can be processed, even unconsciously, and they could contribute to build a multimodal sensorimotor representation of handwriting. This was the rationale when we opted for a ‘natural’ sonification in which the auditory information was quite spontaneous for the listeners: we chose friction sounds that afforded handwriting perception. The vertical pen pressure on the paper was associated to the sound volume and the pen-lifts were rendered by silences. The only sounds which were new for the listeners were the impact sounds associated to the supernumerary peaks (SNvpd) representing the movement dysfluency [28]. Two interpretations are possible for this correct implicit marking. On the one hand, the participants might have referred to a multimodal representation of the graphic movement, a representation which included an auditory component corresponding to the sounds they imagined would have been generated by their own, memorized handwriting. On the other hand, they could have used a totally different strategy, not related to their own experience, but based on esthetic criteria such as ‘the crackling sounds are not pleasant, so they are probably related to a bad handwriting’.

However, the 16 other subjects (belonging to clusters 2 and 3) exhibited a different perceptual judgment under implicit conditions, e.g. they did not use the crackling sounds. Since these sounds had never been heard before the experiment, this is not totally surprising. These subjects did not understand the metaphor that had been suggested to them and decided not to consider these sounds in their evaluation. On the one hand, the 6 subjects belonging to cluster 3 did not use the sounds at all: their correlations were far from significant. Strangely, they positively considered the silences in their scoring. Since they did not know that they were listening to a single word, they might have interpreted the silences as lifts between words within a sentence. On the other hand, the 10 subjects belonging to cluster 2 are also intriguing: their scoring was slightly but correctly correlated to the velocity, whereas they considered the rate in the wrong way under the implicit condition. For these participants, the greater the rate, the lesser the handwriting quality, as if a fast rate was associated with a less legible trace (scribbles for instance). We all know that fast handwriting is not always legible: adults taking notes write at a high speed and might later find it difficult to read what they have written.

In order to identify to what extent the writing sonification was beneficial compared to a standard visual evaluation of handwriting, we conducted the following control experiment on visual examination of the written trace.

## Experiment 3: Handwriting Visual Examination

### Materials and Methods

#### Participants

Sixteen adults (32.8 ± 10.3 years, 8 women) recruited among the staff and the students of the laboratory volunteered for the experiment. None of them had been involved in the previous experiment. They were not experts in handwriting and they were fully naïve about the goal of the experiment.

#### Stimuli

Three of the words written by each writer were randomly selected and printed on a white paper sheet. In all, 45 words were presented to the participants (3 repetitions × 5 writers × 3 groups) The size of the words was standardised. The dot on the letter ‘i’ was removed before printing in order to make the words comparable with the previous experiment.

#### Task

Participants were informed that they would have to mark the quality of the written word on a scale from 0 to 20. They were not told during the whole experiment that they would have to score the handwriting of three different categories of writers. The 45 words were presented in a pseudo-random order for each participant in such a way that no two words from the same writer were placed adjacently. The experiment lasted for between 5 and 10 minutes. The mean scores per category of writers were computed for each participant.

#### Data analysis: Comparison between auditory (Experiment 2) and visual (Experiment 3) evaluation

In order to determine whether the auditory evaluation of handwriting was more efficient for discriminating the three categories of writers than the standard visual evaluation, a first mixed-design ANOVA (type III) was carried out on the mean scores attributed by the 48 participants in the experiment 2 (explicit condition) and by the 16 participants of experiment 3. Two independent variables were controlled: “Modality” (auditory vs. visual) and “Category” of writers (DC, PC and PA). Bonferroni’s post-hoc tests were used when necessary. Finally, in order to test if the writing sonification was beneficial against the established visual methods, a second repeated measures ANOVA was carried out on the score differences between the adjacent categories (PC-DC and PA-PC), both in auditory and visual condition.

### Results

**Fig 3:**
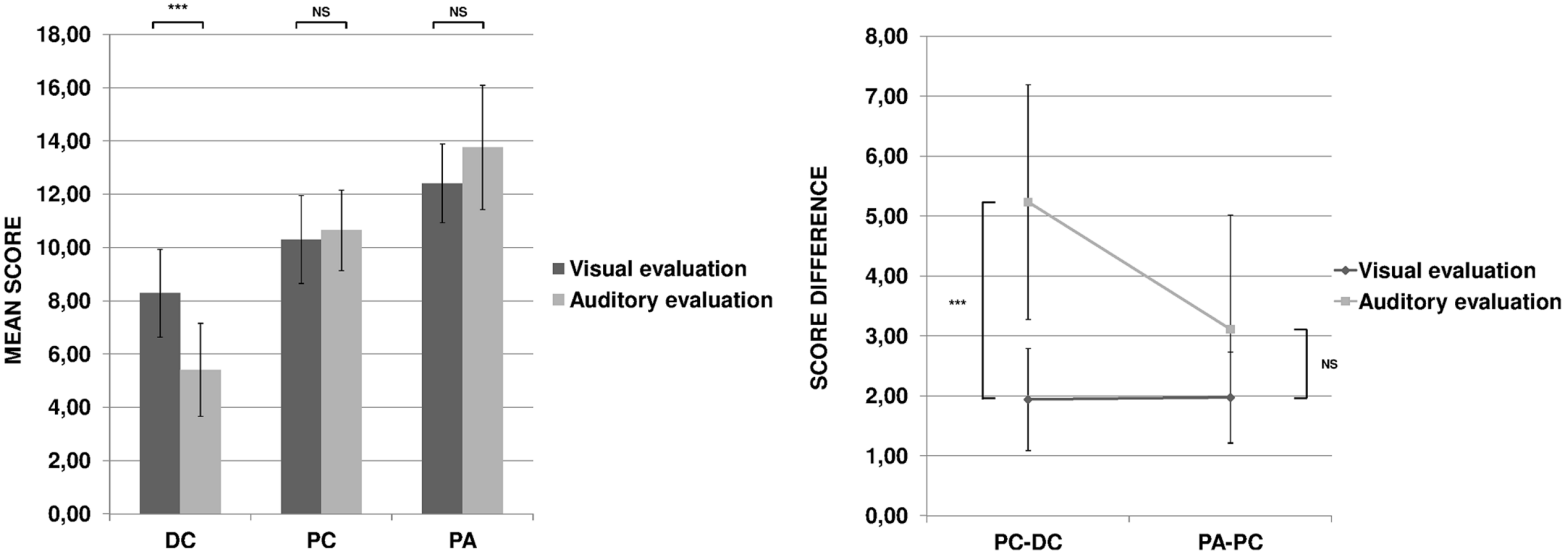
On the left, mean scores attributed to children with dysgraphia (DC), proficient children (PC) and proficient adults (PA) in the visual and auditory evaluations. On the right, score differences between the proficient children and the children with dysgraphia (PC-DC) and between the adults and the proficient children (PA-PC). Error bars correspond to inter-individual variability. NS: not significant; ***: p < 0.001.

The scores given by the participants are within the Supporting Information file S3 Dataset. The results are presented in Fig 3. The first ANOVA on the mean scores revealed no main effect of modality (F(1, 62) = 1.62, n.s.): The overall mean score attributed to the writers was the same in both evaluations. The “Category” factor gave rise to a significant effect (F(2, 124) = 213.87, p < 0.001, *η*_G_² = 0.59). The post-hoc tests of the main effect showed that, for both auditory and visual modality, the scores attributed to the three categories of writers were significantly different. The “Modality” by “Category” interaction was also significant (F(2, 124) = 29.80, p < 0.001, *η*_G_² = 0.17). The post-hoc tests of this interaction revealed that the scores in auditory and visual evaluation were not different except in children with dysgraphia where they were significantly lower in the auditory evaluation (Fig 3, left).

The second ANOVA on the score differences showed a main effect of “Modality” (F(1, 62) = 34.65, p < 0.001, *η*_G_² = 0.24): the score differences between the adjacent categories (PC-DC and PA-PC) was greater in the auditory than in the visual evaluation. The effect of “Adjacent categories” was also significant (F(1, 62) = 10.18, p < 0.01, *η*_G_² = 0.07). Finally and more importantly, the interaction between “Modality” and “Adjacent categories” was significant (F(1, 62) = 10.88, p < 0.01, *η*_G_² = 0.07). As can be seen in Fig 3 (right) and as confirmed by the post-hoc tests, only the difference in score attributed to PC and DC was significantly higher in auditory than in visual evaluation (p < 0.001).

### Discussion

As expected, judging the quality of the handwriting by visual inspection was possible. Furthermore, in spite of the fact that the participants were different and less numerous in this experiment, the overall mean score attributed to the writers was the same as in the previous experiment, when the evaluation was based on sounds. As in the previous experiment, the words written by children with dysgraphia were scored with a lesser value than those written by the proficient children, which were themselves marked lower than those of adults. The comparison of the scores given in the visual and the auditory (explicit) experiments showed that the dysgraphic handwriting was evaluated with a lower score in the auditory condition than in the visual condition. The mean score given to the proficient children was quite similar in the two conditions and the difference between the adjacent categories of children (DC-PC) was greater for the auditory than for the visual condition.

The visual evaluation of handwriting was based on the legibility of the letters composing the word. Of course, such a legibility criterion was unavailable in the auditory evaluation. In other words, the viewers evaluated the word legibility without any knowledge about the underlying movement, whereas the listeners evaluated the handwriting movement without any knowledge on the final legibility. Now, if we may assume that a skilled movement leads to a legible word, the reverse is not true: some children with dysgraphia produce a relatively readable trace but at the price of a slowness and difficulties in writing [21].

## General Discussion

The purpose of the present study was to assess whether translating some handwriting movement characteristics into sounds may improve the evaluation and possibly the diagnosis of handwriting troubles. In a first step, we collected handwriting samples from 15 writers belonging to 3 categories of writers: 5 proficient adults (PA), 5 proficient children (PC) and 5 children with dysgraphia (DC). In a second step, we sonified the collected handwriting samples and created 45 audio files. In the third step, we asked 48 naive listeners to mark the quality of the handwriting that was supposed to have generated the sounds they heard. In order to evaluate the relevance of the sonification strategy, we used two experimental conditions: in the first, “implicit” condition, participants had prior knowledge neither about the meaning of the sounds, nor about the evaluation criteria. In the second, “explicit” condition they knew what the sonified variables corresponded to, how they were sonified, and the evaluation criteria. Hence, they were aware of the signification of the sounds. In a final step, we asked 16 naïve participants to mark the visual quality of the written trace. The aim of this supplementary experiment was to determine whether the auditory modality was better suited for discriminating between the three categories of writers than the visual modality. Importantly, under both auditory and visual conditions, the participants were unaware that they were judging the handwriting of three different categories of writers.

Overall, the results of the auditory experiment confirmed the results of the experiment by Thoret et al. [35] showing that subjects were able to associate a biological movement to sounds whose timbre variations had been generated by velocity profiles following the 1/3 power law. In that study, graphical movements were used although they did not correspond to the handwriting of words. These movements were produced by a fluent writer and they respected the 1/3 power law. In handwriting, particularly among children, this rule is not always perfectly respected [41] and the close visual control exerted by children strongly changes their kinematics [42–44]. Therefore, the present study extends these results to real handwriting.

If sounds enable external adult listeners to discriminate between fluent and poor handwriting, the sonification of handwriting might be useful for helping the diagnosis of dysgraphia. The two groups of children whose handwriting was compared here were constituted on the basis of their BHK scores. These children appeared to have both different handwriting traces and movements. However, the results of the visual experiment confirmed that visual examination of the static written trace cannot easily show difficulties in movement. Objective digitizer-based analyses enable the capture of information on handwriting dynamics, providing data beyond that which is observable to the human eye [45]. Handwriting sonification could therefore be a supplementary tool, comparable to the stethoscope, allowing the therapist to hear the handwriting movement and to estimate by ear whether these movements are correct. The aim of sonification is not to replace the classical visual assessment of handwriting, but to improve it by making possible to perceive new features of handwriting which are hidden to the therapist and which can be useful for the diagnosis of dysgraphia. The static trace (handwriting quality) might be visually analyzed with the classical BHK items, whereas handwriting fluency (movement quality) might be perceived by ear. Furthermore, real-time sonification of handwriting would allow the therapist to capture when the movement is not fluent and to link it to the simultaneous visual inspection of the quality of the written trace or of the children posture during writing. Therefore, coupling the BHK test and the sonification would allow for the assessment of both the trace and the handwriting movement.

Beyond helping for the dysgraphia diagnosis, handwriting sonification could be very helpful for rehabilitation. The same sonification strategy could indeed be employed to provide real-time auditory feedback to children, helping them to perceive incorrect movements. Several attempts have already been made supplying a writer with feedback related to his/her handwriting movements. Haptic guidance, by means of a robot, has for instance been tested [46]. The authors obtained a positive effect when the difference in acceleration between the ideal and the actual trajectories was controlled by the robot. However, kinesthetic perception relies on a proprioceptive integration that children are not always capable of [43]. It could be relevant to find other ways to make handwriting kinematics perceivable. Using real-time visual feedback has also been tested, but it tends to maintain both slowness and dysfluency in handwriting [47]. We think auditory feedback might be an interesting possibility. Although several constraints imposed by the real-time sonification are to be overcome, preliminary promising results have already been obtained and work is currently in progress [48, 49, 50].

## Supporting Information

S1 Audio Sonified handwriting of a child with dysgraphia. (WAV)

S2 Audio Sonified handwriting of a child with a proficient handwriting. (WAV)

S3 Audio Sonified handwriting of an adult with a proficient handwriting. (WAV)

S1 Dataset Experiment 1. (ZIP)

S2 Dataset Experiment 2. (TXT)

S3 Dataset Experiment 3. (TXT)
